# Exopolysaccharides: promising applications in bioremediation for waste removal

**DOI:** 10.3389/fmicb.2026.1859365

**Published:** 2026-06-24

**Authors:** Saurabh Gangola, Samiksha Joshi, Ashish Kumar, Shivanshu Garg, Narendra Singh Bhandari, Rajeshwari Lekhwar, Kumar Gaurav, Anuj Saraswat, Pratheesh Prakasam Thanka, Sunil Kumar, Prashant Kumar

**Affiliations:** 1 Department of Microbiology, Graphic Era (Deemed to be University), Dehradun, India; 2 School of Biosciences, Swami Rama Himalayan University, Dehradun, India; 3 Department of Microbiology, GBPUA&T, Pantnagar, India; 4 Department of Biochemistry, Lovely Professional University, Phagwara, Punjab, India; 5 Faculty of Agricultural Sciences, Shree Guru Gobind Singh Tricentenary University, Gurugram, Haryana, India; 6 School of Agriculture, Graphic Era Hill University, Dehradun, India; 7 Department of Biology, College of Science, United Arab Emirates University, Al Ain, Abu Dhabi, United Arab Emirates; 8 Department of Electronics and Communication Engineering, Teerthanker Mahaveer University, Moradabad, India

**Keywords:** biodegradation, biosorption, environmental pollution, heavy metal removal, sustainable environment, wastewater treatment

## Abstract

Waste management is one of the global prime concerns in the 21st century. Due to the increasing human population, industrialization, and urbanization, a huge amount of waste is generated, posing severe environmental and public health risks. Conventional methods of contaminants removal are neither cost-effective nor efficient. Therefore, there is an urgent need to adopt biological strategies to effectively degrade and inactivate toxic contaminants. Microbial bioremediation for contaminant removal is a cost-effective and environmentally safe technology in which microbial biomass and its products degrade or convert more toxic substances into less toxic substances. Biopolymers produced by microorganisms can absorb contaminants, thus providing an excellent tool for bioremediation. Microbial exopolysaccharides (EPS) are complex and high molecular weight polymeric substances that are chemically composed of polysaccharides, proteins, lipids, humic substances, and uronic acids. EPS of microbial origin are ideal for bioremediation as they display excellent physiological, rheological, and physicochemical properties. Exploitation of microbial EPS for bioremediation is increasing, and new interventions are continually sought to enhance its biodegradation effectiveness. This review article explicates the potential of microbial EPS for bioremediation of contaminants in aquatic and terrestrial ecosystems in a highly economical, sustainable, and eco-friendly manner. It also highlights the current challenges and prospects for making EPS-based applications efficient in bioremediation for waste removal.

## 1 Introduction

Exopolysaccharides (EPS) are primary macromolecules and complex biopolymers in microbial collections with high molecular weight majorly composed of lipids (Rhamnolipids, Trehalolipids, Mannosylerythritol lipids, Surfactin, Mycolic acids, Phospholipids, Glycolipids, Lipopolysaccharides, Ergosterol), sugars (homopolysaccharides (d-glucose, d-fructose, d-mannose, l-rahmnose, d-glucoronic acid, d-galacturonic acid)) and heteropolysaccharides (Xanthan, gellan, Dextran, Levan, Hyaluronic Acid, Clavan), proteins, or humic acids (Rana and Upadhyay, 2020). EPS of microbial origin are more beneficial than others, such as glycogen, starch, carrageenans isolated from animals, plants, and algae, respectively. Bacterial EPSs are diverse in structure and are divided into loosely associated slime layers and closely associated capsular polysaccharides (Xiao et al., 2022). EPS are basically of two types: homopolysaccharides, made up of a single type of monosaccharide, either branched or unbranched, for example, D-glucans or fructans (levan and inulin), and heteropolysaccharides, composed of two or more units of different monosaccharides, for example, hyaluronic acid, xanthan, kefiran, alginate, and gellan (Mohd Nadzir et al., 2021). Bacteria use four different mechanisms for the synthesis of these polymers, including extracellular synthesis via a sucrase- and synthase-dependent pathway commonly used for the synthesis of homopolysaccharides, while the ABC transporter-dependent pathway and the Wzx/Wzy-dependent pathway are used for heteropolysaccharides production (Schmid et al., 2015). Some of the most commonly known genera that produce EPS include Acetobacter, Rhizobium, Gluconobacter, Klebsiella, Gluconacetobacter, Agrobacterium, Zymomonas, Azotobacter, Alcaligenes, Xanthomonas, Pseudomonas, Enterobacter, Alteromonas, Halomonas, Bacillus, Streptococcus, Paenibacillus, and others (Díaz-Cornejo et al., 2023). EPS is an inherent component of microbial biofilms that serve many functions such as surface attachment, bio-aggregate formation, colonization and nutrient uptake and provides protection to the host bacterium against salinity, temperature, aridity, desiccation, antimicrobial antibiotics as well as drugs during unfavorable conditions (Casillo et al., 2018; Merino et al., 2019). Microbially produced EPSs are non-toxic, viscoelastic, pseudo-plastic, thixotropic, biocompatible and biodegradable polymer with outstanding applications in different industries (Siddharth et al., 2021) as well as efficient in bioremediation and degradation of long-chain n-alkanes, oil-contaminated fields and petroleum hydrocarbons (Poli et al., 2010). Waste water treatment using EPSs has shown significant results for removal of heavy metals, dyes, petroleum products, pharmaceutical compounds (Shi et al., 2022) EPS secreted from extremophiles are gaining special attention for waste water treatment as they are rich in charged anions like residues of uronic acid and sulfates that work as ligands for scavenging heavy metals and toxic compounds from wastes (Lo Giudice et al., 2020). So, the extraction techniques for EPS are much needed. The commonly used extraction methods include microwave-assisted, enzyme-assisted, ultrasound-assisted, and hot-water-assisted. EPS in wastewater treatment (WWT) systems, especially in the activated sludge process, has made a significant contribution by influencing the properties of microbial floccules, including hydrophilicity, surface, aggregate stability, transferability, and hydrophobicity (Zhao et al., 2019). Therefore, the spatial distribution of EPS directly influences the bioflocculation, dewatering, and settling of activated sludge (Zhang et al., 2015). As EPS have the potential to mass transfer between cells and environments, they can affect pollutant removal and microbial metabolism (Zhao et al., 2019). One of the most important strategies microbes use to resist heavy metal stress is EPS synthesis. EPS have numerous functional groups, most of which are anions at neutral pH and capable of forming organometallic complexes with metal ions. Therefore, bacterial EPS also act as biosorbents for the removal and recovery of heavy metals from certain industrial wastewater (Sheng et al., 2010). Among microorganisms, intact microbial cells as well as cell-bound EPS from fungi, microalgae, and bacteria have been scientifically proven to be EPS producers and have a wide range of applications for the remediation of heavy metals, dyes, pharmaceutical compounds, and petroleum products in environmental waste and industrial water sources. The binding of heavy metals to EPS occurs through different processes, such as ion exchange, complexation, and surface micro-precipitation. Binding affinity is decided by the number of binding sites on the surfaces or pores of EPS flocs and the composition of EPS. Heavy metals form organo-metal complexes at an optimum pH by hydrophilic interaction between HMs and carboxylic or phosphoric groups of EPS (Sheng et al., 2010). Additionally, hydrophobic interactions help embed the heavy metal. The adsorption efficiency depends on the types of EPS fractions and properties of the heavy metals (Wei et al., 2017). EPS is produced in a harsh environment under heavy stress and different nutritional conditions (Mohite et al., 2017). Due to its acidic nature, EPS can tolerate heavy metal stress and is therefore preferable to heavy metal removal (Bhunia et al., 2018). The diverse structure of bacterial EPS, owing to the presence of various functional groups (acetate, carboxyl, hydroxyl, carbonyl, etc.), enables their modifications to introduce additional valuable properties. Advanced techniques in genetic and metabolic engineering, under optimal cultivation conditions, can modulate the yield and the structural and functional properties of bacterial EPS (Xiao et al., 2022). Recognizing EPS’s outstanding ecological and commercial value, bacterial EPSs have been projected to be a cost-effective, sustainable, and straightforward alternative to economical bioremediation of the environment. In the present review, different strategies for the use of EPS in bioremediation, biosorption, and bioaccumulation of heavy metals, wastewater treatment, the role of extremophilic EPSs in bioremediation, and recent nanobiotechnological advances are discussed. This review adopts a semi- systematic narrative approach to critically evaluate the role of microbial EPS in bioremediation. Relevant literature published between 2000 and 2026 was collected from databases including Scopus, Web of Science, PubMed, and Google Scholar using keywords such as microbial EPS, biosorption, bioremediation, heavy metal removal, and wastewater treatment. In addition to summarizing organism- and pollutant-specific studies, this review comparatively discusses adsorption mechanisms, pollutant removal efficiencies, and environmental fate of EPS-bound contaminants to provide a more comprehensive understanding of EPS-based remediation technologies.

## 2 Biosynthesis of EPS in a toxic environment

Biosynthesis of EPS depends on the mode of mechanism involved or the site of synthesis (extracellular or intracellular). Synthesis of carbohydrate polymers in bacteria occurs mainly by four different mechanisms: (i) ABC transporter-dependent pathway; (ii) Wzx/Wzy-dependent pathway; (iii) extracellular synthesis using sucrase, and (iv) the synthase-dependent pathway. Various enzymatic conversions take place inside the cell and follow the same process of producing activated sugar acids or sugars using the precursor molecules for stepwise elongation of the polymer strand in synthase-dependent, ABC transporter-dependent, and Wzx/Wzy-dependent pathways, whereas in the case of the extracellular pathway, direct addition of monosaccharides is observed by breakdown of disaccharides or trisaccharides for elongation of the polymer strand. In the Wzx/Wzy-dependent pathway, undecaprenol diphosphate (C55), an anchor at the inner membrane, is linked to individual repeating units assembled by glycosyltransferase enzymes and translocated across the cytoplasmic membrane by a Wzx protein also known as flippase. After that, polymerization is carried out by the Wzy protein in the periplasm before being exported to the cell surface (Islam and Lam, 2014). Final transport of the polymer from the periplasmic space to the cell surface depends on polysaccharide co-polymerase and the outer membrane polysaccharide export families (Morona et al., 2009). The Wzx/Wzy pathway is known mainly for the synthesis of polysaccharides with diverse sugar patterns and is generally classified as a heteropolymer pathway, producing polymers such as xanthan. Microbial strains that follow this extracellular polysaccharide pathway carry the Wzx (flippase) and Wzy (polymerase) genes in their operons. Another pathway is the ABC transporter-dependent pathway, mainly found in bacteria that produce CPS (capsular polysaccharide) (Whitney and Howell, 2013). Synthesis of CPS via ATP-binding cassette transporter-dependent pathway involves assemblage using glycosyltransferases at the inner membrane, leading to formation of homopolymers by a single GT or in heteropolymers by multiple GT’s (Whitney and Howell, 2013). The export process across the membrane involves a tripartite efflux pump-like complex composed of ABC transporters and periplasmic proteins from the polysaccharide co-polymerase (PCP) and the outer membrane polysaccharide export (OPX) family (Morona et al., 2009). The synthase-dependent pathway is independent of flippase enzyme for the translocation of repeating units and secretes a complete polymer across the membranes and the cell wall. Furthermore, the synthase protein is responsible for both polymerization activity and the translocation process (Rehm, 2010). Synthase-dependent pathways are primarily used for the production of homopolymers from a single sugar precursor, for example, curdlan composed of only *β*-(1–3)-linked glucose, or cellulose composed of β-(1–4)-linked glucose units. Hyaluronic acid (HA) synthesis is catalyzed by hyaluron synthase, which carries out both secretion and polymerization. A disaccharide is composed of two precursor molecules, N-acetylglucosamine and glucuronic acid (Chong et al., 2005). Therefore, HA synthesis differs from other synthase-dependent pathways. In exopolysaccharide biosynthesis, maximum enzymatic steps occur inside the cell while secretion or polymerization process is carried out in the cell envelope. However, in few cases of extracellular synthesized polysaccharides, like levan or dextran biosynthesis occurs via glycosyltransferases which are covalently linked or secreted to the cell surface. Homopolysaccharides like levan, mutan, and dextran are synthesized extracellularly, where levan sucrase and dextran sucrase, as enzymes, and sucrose as a substrate are involved in transferring the activated precursor monosaccharides from substrate to growing polysaccharide. The polysaccharides then assemble in a specific pattern with particular branching and linkage. This type of synthesis is commonly reported in Gram-positive cocci (Whitfield, 1988). Conversely, intracellular synthesis of either a homopolysaccharide or a heteropolysaccharide is a complex process. It involves the assembly of sugar nucleotide precursors, after which the resulting molecules are exported from the cell. The process is carried out by several enzymes and regulatory molecules. Any form of sugar enters as a substrate, actively or passively, in a bacterium, and further gets catabolized by intracellular phosphorylation or periplasmic oxidation (Freitas et al., 2011). The intracellular machinery requires charged and energy-rich precursors in the form of nucleotide diphosphate/monophosphate. Furthermore, the phosphorylated sugar (1P/sugar-2P or 6P) serves as an activated primary residue (Madhuri and Prabhakar, 2014). Finally, different intermediates obtained from metabolic pathways like Glucose-1P or Fructose-6P lead to the synthesis of Uridine di-phosphate-N acetyl galactosamine, dTDP rhamnose, and Uridine di-phosphate-N acetyl glucosamine precursor molecules for EPS synthesis (Boels et al., 2001). The enzymes responsible are Phosphoglucomutase enzyme, UDP-glucosepyrophosphorylase, and dTDP-glucosepyrophosphorylase for the conversion of sugar-6P to sugar-1P, sugar-1P to UDP-glucose, and dTDP-glucose, respectively (Madhuri and Prabhakar, 2014). In *Lactobacillus rhamnosus*, Uridine di-phosphate -N-acetylglucosamine 4 epimerase catalyzes the transformation of UDP-GlcNAc to UDP-GalNAc (Boels et al., 2001). Furthermore, some genes have been reported to be involved in the synthesis of dTDP-L rhamnose NDP-sugar molecule in *Lactobacillus rhamnosus* strains. Thereafter, enzyme glycosyltransferase transfers sugar molecules from activated NDP/NMP-sugar moieties to (C55-P), undecaprenyl phosphate, an isoprenoid lipid carrier located at the cytoplasmic membrane (Sutherland, 2001). According to several reports, six different glycosyltransferase (GT) genes are responsible for the assembly of heptasaccharide units with rhamnose moieties in *L. rhamnosus* (Boels et al., 2001). Such a lipid intermediate pathway has also sometimes been utilized for Gram-negative bacterial EPS for assembly and transport. However, Gram-negative bacteria mostly follow either the Wzx-Wzy dependent or the ABC transporter dependent pathway (Freitas et al., 2011), with a few exceptions like *P. aeruginosa* following the Synthase-dependent pathway. *E. coli*-k, a Gram-negative bacterium, has been observed to assemble and transport its capsular polysaccharide through this mechanism (Whitfield, 1988). Some evidence has also been obtained for EPS biosynthesis carried out solely from NDP-sugar monomers and without lipid intermediates, in the inner cytoplasmic region, for example, synthesis of cellulose by *Acetobacter xylinum* (Aloni et al., 1983). Post-synthetic modification occurs in the case of homopolysaccharides once they are translocated to the cell surface via enzymatic modification. One such example is alginate production by *A. vinelandii* in which the mannuronic acid residue from the polymannuronic acid molecule at cell surface is converted by mannuronan 50 epimerase to glucuronic acid (Whitfield, 1988). Finally, the fully assembled and exported EPS either attaches to cell surface as capsular polysaccharide or is secreted as slime. The properties and chemical structure of the final polymer vary depending on the biosynthetic pathway. The most commonly involved genes in these biosynthetic pathways include branching enzymes, various glycosyltransferases, polymerizing enzymes, and modification enzymes. Enzymes that encode these functions are present in most of the EPS producing microbes (Rehm, 2010). Despite numerous reports on gene clusters responsible for exopolysaccharide biosynthesis, the modes of action and functions of most genes and proteins remain unknown. Microbes isolated from stressed conditions counteract toxicity via different mechanisms like extrusion, biotransformation, detoxification, and EPS production (Bourles et al., 2020). Amongst all, EPS production by bacteria is one of the best ways to survive under unfavorable conditions like nutrient deficiency, fluctuation in temperature, pH, and the presence of toxic compounds (Poli et al., 2010). To survive under metal stress conditions, the bacteria produce large quantities of exopolymeric substances (Ibrahim et al., 2020) with several anionic functional groups, such as carboxyl, sulfate, hydroxyl, phosphate, and amine groups in the structure that can bind with metal ions. Various investigations showing strong affinity of uranium ions with amino, carboxylic, or hydroxyl groups of EPS (Kazy et al., 2009) and high metal adsorption potential of EPS (Iyer et al., 2005) for metal contaminant removal from the polluted environment have attracted prominent attention because of their biodegradable properties and eco-friendly nature (Zhao et al., 2018). Furthermore, for the remediation of heavy metals, EPS biosynthesis mainly incorporates anionic functional groups to produce negatively charged EPS as a suitable biosorbent. Few bacterial species used for the synthesis of anionic EPS include *P. aeruginosa, A. vinelandii* for alginate; *Enterobacter* A47 for fucopol, *Sphingomonas paucimobilis* for gellan; *Xanthomonas campestris* for xanthan; *P. oleovorans* for galactopol; and *Pasteurella multocida*, *P. aeruginosa* for hyaluronan (Öner, 2013). Furthermore, the adsorption efficiency of EPS for heavy metals is affected by constituents of EPS, temperature, pH, concentration of adsorbent, and concentration of adsorbate (Wei et al., 2016). Various abiotic factors are found to be interlinked with biosynthesis of EPS such as factors like temperature, pH, and growth pattern of bacteria can alter the function group, branching, chain length of polymer, structural complexity, non-carbohydrate substituent, diverse bondings and arrangement of side chains. The key pathways of EPS biosynthesis remain the same in normal and toxic environments; however, the toxic environment may trigger the overexpression of genes such as *wzx, wzy*, and glycosyltransferases as well as stress-response genes, which lead to increased EPS production and composition changes for microbial protection (Schmid et al., 2015).

## 3 Types of pollutants remediated by EPS

EPS have been known for their bioremediation potential since the 1960s, as they can absorb, remove, or convert various toxic pollutants into nontoxic forms. Bioremediation of hazardous heavy metals using EPS is proven to be cost-effective, safe, low energy consuming and environmentally friendly approach (Gutierrez et al., 2008). Removal of different heavy metals has been reported via adsorption by EPS of *Pseudomonas pachastrellae, Bacillus cereus, Mesorhizobium loti, Bacillus firmus, and Cryptococcus laurentii* (Du et al., 2012). Furthermore, some other studies showing removal of different metal ions through biosorption or biomineralization include fucose-containing polysaccharide secreted by *Enterobacter A47* for lead removal, *Arthrobacter viscosus* for cadmium removal, and *Halomonas* sp. *Exo1* for arsenic removal*, Bacillus thuringenesis* for mercury removal, and *Enterobacter cloacae* for chromium removal (Kumawat et al., 2021). EPS of *Sphingomonas* sp. *MKIV* has been reported to remediate 90% of the ionic liquids rich in organic/inorganic anions and nitrogen-containing cationic aromatic rings from wastewater effluent (Koutinas et al., 2019). In other reports, metallic wastewater contaminants are efficiently treated with electroactive bacteria such as *Shewanella oneidensis* for uranium via sorption followed by reduction, *Pseudomonas putida* for arsenate via reduction, and dibenzothiophene via biotransformation. EPS (heteropolymer) released from Streptomyces sp. showed 93% removal of Sr^2+^ from radionuclide solution (Koutinas et al., 2019). EPS producing *Bacillus* sp. isolated from tannery activated sludge showed effective removal of Cr (VI) (Zhu et al., 2019) and *Pseudomonas* sp. W6 isolated from an Indian hot spring showed biosorption and effective removal of Pb from synthetic wastewater (Kalita and Joshi, 2017). A few other bacteria, such as *L. plantarum-605, B. cereus* KMS3-1, and *Deinococcus radiodurans R1,* interact with metal ions to assist the detoxification process and efficient sorption of different heavy metals, such as lead, cobalt, nickel, copper, cadmium, and synthetic dyes like methylene blue (Mathivanan et al., 2021). Furthermore, polluted water contaminated with different colorant residues coming from textile industries can be treated by bioremediation using microbial EPS, such as azo dyes (congo red and methyl orange) degraded by EPS-stabilized silver nanoparticles synthesized by *Leuconostoc lactis*, carcinogenic malachite green removal by halophilic bacterium *Exiguobacterium* sp. VK1, remazol blue removal by *Ochrobactrum* sp. and *P. aeruginosa*, Azo dyes by *Aliiglaciecola lipolytica* methylene blue removal by *lactobacillus plantarum* using hetero exopolyssachride (de Oliveira et al., 2018). Bioremediation of contaminated petroleum sites and oil spills has also been reported using EPS produced by *Enterobacter cloacae, Halobacillus* sp., and *Sporosacina halophila* (Kumawat et al., 2021). Few studies demonstrated the breakdown of toxic chemicals by microbial EPS, such as EPS from *Klebsiella* sp., degrading toluene, n-hexadecane, olive oil, and kerosene; *P. putida* for breakdown of different hydrocarbons, *Aspergillus niger* and *Zoogloea* sp. for degradation of pyrene, a polycyclic aromatic hydrocarbon, *Enterobacter cloacae* degrading polycyclic aromatic hydrocarbons (PAHs) and alkanes (Harish et al., 2012). An alkaliphilic bacterium, *Cronobacter sakazakii*, isolated from oil-contaminated wastewater showed emulsifying activity against aromatic and aliphatic hydrocarbons (Jain et al., 2012). EPS from *Thauera* sp. are reported to have effective properties for the biodegradation of acetone, bio-plugging, oil emulsification, and biodegradation of isopropyl alcohol in wastewater. Vandana and Das (2021) demonstrated potential of EPS-forming microbes as bio-emulsifier for bioremediation of petroleum-polluted sites based on the degradation by *Pseudomonas furukawaii* PPS-19 of crude oil within 5 days isolated from the oil-contaminated site. Several reports are available on environmental remediation by EPS from different microorganisms to control synthetic pollutants (Bhatt et al., 2021), heavy metal (Lee and Cho, 2025), radionucleotides (Marques, 2018), xenobiotic compounds (Jeong and Choi, 2020), plastics and various agrochemicals (Kour et al., 2021), and organic pollutants (Kaushik et al., 2021) (Table 1). The iron-reducing bacterium *Geobacter sulfurreducens* and microbial EPS synergistically promoted ferrihydrite transformation, enhancing arsenate immobilization. EPS facilitated mineral phase changes and arsenic binding, thereby reducing arsenic mobility in contaminated environments (Wang et al., 2026). EPS-producing microbes such as Geobacter, Shewanella, and Pseudomonas enhance microbial fuel cell performance by improving biofilm formation and extracellular electron transfer. Their exopolysaccarides also facilitate heavy metal bioremediation, wastewater treatment, sustainable agriculture, green nanoparticle synthesis, and biomedical applications (Singh et al., 2026).

**Table 1:** Types of pollutants remediated by microbial exopolysaccharides.

S. no	Pollutants type	EPS producing microorganism	Representative pollutants	EPS interaction mechanism	Optimal conditions	Absorption	Key functional groups	References
1	Heavy Metals	*Pseudomonas* sp. *H7, Agrobacterium* sp. *Z22*	Pb, Hg, Cr	Ion exchange, precipitation, electrostatic interactions, complexation	pH = between 5.0 and 6.0 Temperature = 32 °C to 37 °C	between 80 and 91%	–COO^−^, –SO₃^−^, –PO₄^2−^, –NH₃^+^	Vandana et al. (2023)
		*Halomonas nitroreducens*	Cd, As	Assists in biosorption and the reduction of toxic metal ions	pH = between 5.0 and 10.0 Temperature = 20 °C to 35 °C	70 to 98%	COO-, NH₃^+^	Banerjee et al. (2021)
		*Sinorhizobium meliloti*	Ni, Cu	Metal sequestration through complexation and selective chelation	weakly acidic conditions		Carboxyl, hydroxyl, amid	Pirhanov (2021)
		*Streptomyces* sp.	Radioactive elements (e.g., Uranium)	Adsorption and immobilization of radioactive ions within the biofilm matrix	pH = 3 to 5	380 *μ* per mol of uranium per gram	Sulfhydryl, Phenolic	Zhao et al. (2022)
		*Paenibacillus polymyxa*	Cu, Pb	Chelation and reductive transformation of heavy metals	pH = 6 to 7.5	85 to 100% for Pb, 75 to 90% for Cu	Thiol, amide	Mohite et al. (2017)
		*Micrococcus luteus*	Cadmium	Cadmium accumulation through cellular encapsulation and uptake	pH = 5.0 to 7.0 Temperature = 30 °C to 37 °C	50% to over 95%	NH₂, PO₄^2−^, SH	Munir Ahamed et al. (2024)
		*Serratia marcescens*	Hexavalent chromium (Cr VI)	Reduction of Cr VI to the less toxic Cr III state	pH = 6.0–8.0 Temperature = 30 °C to 37 °C	60 to 95% reduction	OH, COOH, SH	Chakraborty and Mondal (2024)
2	Antibiotics	*Pseudomonas*, *Bacillus*, *Acinetobacter*, *Shewanella*	Azithromycin, Ciprofloxacin	Adsorption acts as a physical barrier, facilitating antibiotic degradation	pH = 4–6	75 to 98%	Hydroxyl groups, tertiary amides	Acharya et al. (2026)
3	Hydrocarbons	*Alcanivorax*, *Rhodococcus*, *Bacillus*	Aliphatic, aromatic compounds	Emulsification, solubilization, sorption and binding, formation of bio-aggregates	Temperature = 25 °C to 30 °C	80 to 97%	Alkanes, alkenes, alkynes, and aromatics	Li et al. (2023) and Parthipan et al. (2017)
4	Pesticides and Herbicides	*Pseudomonas*, *Bacillus*, *Acinetobacter*, and *Arthrobacter*	DDT, Atrazine, organophosphate	Bioremediation, biosorption, microbial protection	pH = < 6.0	10–40%	Sulfonylureas, thiocarbamates, thiophosphates	Kosimov et al. (2025)
		*Kosakonia oryzae*	Atrazine, glyphosate	Adsorption and enzymatic degradation of pesticides	Temperature = 30 °C to 32 °C	10–30%	OH, COOH, NH₂, SH, PO₄^2^	Dash and Osborne (2020)
5	Organic Pollutants	*Acinetobacter*, *Rhodococcus*, and *Arthrobacter*	Polychlorinated biphenyls, polybrominated diphenyl ethers, chlordane.	Electrostatic forces, hydrophobic interactions, and biotransformation	Temperature = 28 °C to 30 °C, pH = 7.0 to 7.5	70–90%	Halogenated compound, oxygen-containing, nitrogen-containing groups	Martínez (2024)
6	Synthetic Dyes	*Enterobacter*, *Shewanella*	Azo dyes, Anthraquinone dyes	Electrostatic interaction, hydrogen bonding, static quenching	Temperature = 35 °C to 37 °C, pH = 7.0 to 8.0	85% to over 99%	Azo, hydroxyl, amino, sulfonic acid	Yue et al. (2025)
		*Acinetobacter* sp.	Industrial dyes	Binding and molecular entrapment of dyes for effective removal	pH = 6.0 to 9.0	74% to over 98%	NH₃^+^, NH₂ (protonated amines), Quaternary ammonium (NR₄^+^), OH₂^+^	Datta et al. (2020)
7	Nanoparticles	*Aspergillus*, *Penicillium*, *Trichoderma*	Titanium dioxide, silver, zinc oxide, and iron oxide	Cation bridging, hydrophobic interactions	pH = slightly acidic to neutral conditions, Temperature = 25 °C to 37 °C		Amine, hydroxyl, carboxyl	Li et al. (2020)

Although several studies have demonstrated the effectiveness of microbial EPS in pollutant remediation, comparative evaluation capacity, kinetic behavior, optimal conditions and pollutant-specific efficiencies remains limited. Therefore, a comparative summary of representative EPS-mediated remediation studies, including adsorption capacity, pH conditions, and adsorption/isotherm models, has been incorporated to provide better mechanistic and quantitative insights into the EPS-based bioremediation system.

Most EPS-mediated adsorption systems followed Langmuir or the Freundlich isotherm behavior, indicating both monolayer and heterogeneous adsorption processes. Similarly, pseudo-second order kinetics were frequently observed, suggesting chemisorption as the dominant mechanism. Pollutant removal efficiency was strongly influenced by pH, temperature, ionic strength, and the availability of functional groups in EPS, such as carboxyl, hydroxyl, sulfate, and phosphate groups.

## 4 EPS-mediated biodegradation of waste

**Figure 1:**
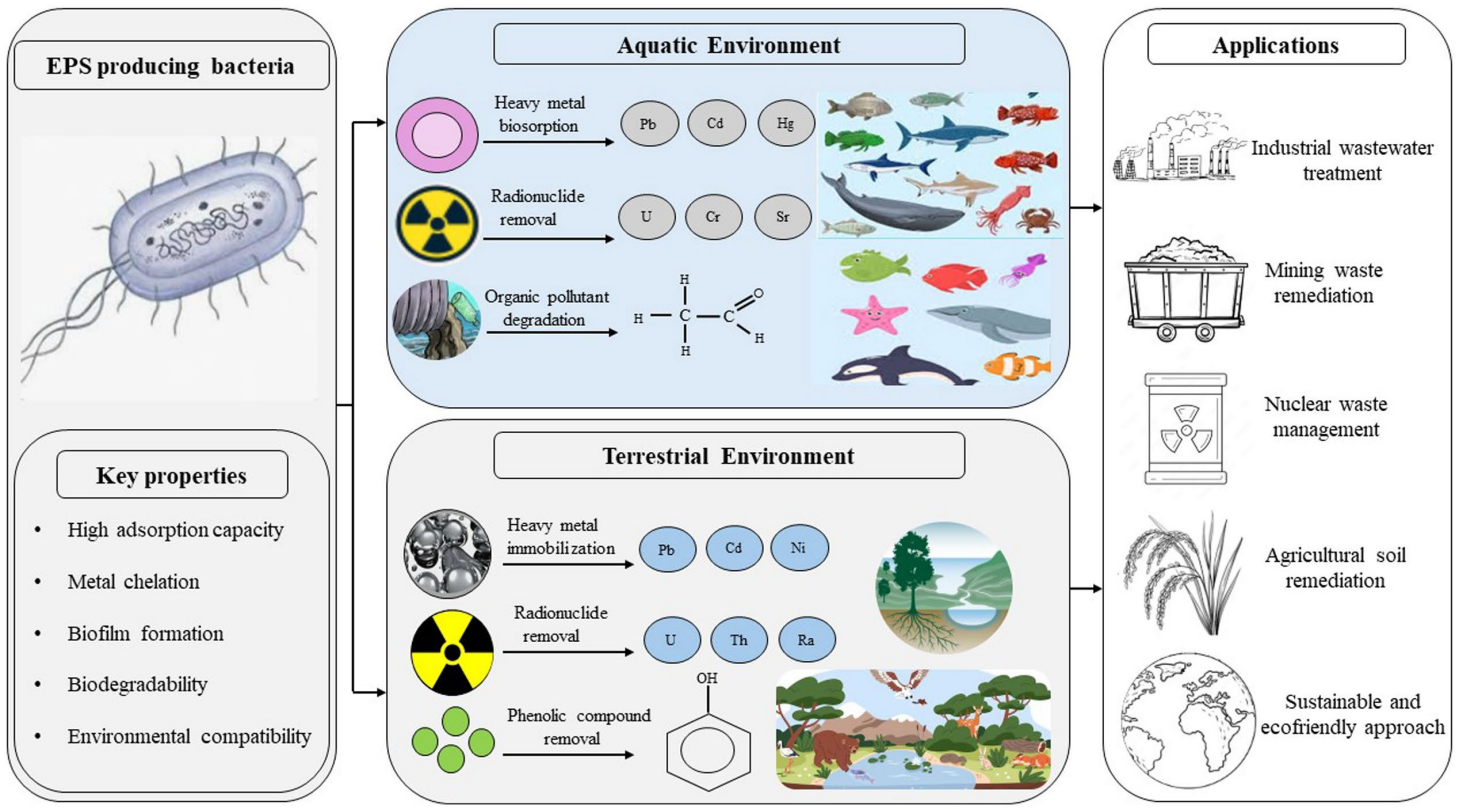
Role of microbial EPS in bioremediation of aquatic and terrestrial environments by removal, adsorption, and immobilization of heavy metals, radionuclides, and organic pollutants for sustainable environmental applications.

Microbial EPS have a substantial role in restoring, degrading, and removing metals, specifically from the harmful industrial waste. Because of its acidic structure and non-carbohydrate composition, EPS provides environmental resilience to heavy metal stress (Ghosh et al., 2024). Additional functional groups that exhibit intrinsic affinity for metal ions include carboxyl, carbonyl, amide, amine, imidazole, imine, hydroxyl, phosphodiester, sulfonate phosphonate, sulfhydryl and thioether found in EPS (Sreedevi et al., 2022). Moreover, it will increase uronic acid concentration lead to enhance its anionicity, which is crucial for interaction and binding of important cationic heavy metals such as Hg^2+^, Ni^2+^, Cd^2+^, Pd^2+^, Zn^2+^, Cr^2+^, etc. This particular characteristic makes microbial EPS suitable for use as a biological agent for biosorption-based metal removal (Pagliaccia et al., 2022). The types of functional ionic groups on the surface of EPS, the bioavailability of metal ions, the pH of the ecosystem, and the microbial consortia that make EPS are some of the multiple variables that influence the interaction between EPS and metal ions. Thus, the mechanism and metal-binding characteristics are crucial for reducing the risks of harmful metal mobility and bioavailability via immobilization, metabolic, or non-metabolic accumulation (Fakhar et al., 2022). Therefore, as a possible biosorbent, EPS-mediated bioremediation enables both the removal and recovery of metals from the waste products at both terrestrial and aquatic landscapes (Figure 1). There are several biosorption processes that primarily employ EPS to sequester metals. The two techniques that use polysaccharides for biosorption are complexation and ion exchange (Munir Ahamed et al., 2024). Complexation occurs when metal ions combine with carboxyl groups (-COOH) that are present in microbial polysaccharides to create a complex on the cell surface, removing the metallic ions from an aqueous solution. The cell walls of microorganisms contain polysaccharides that participate in ion exchange. These contain K^+^, Na^+^, Ca^2+^, and Mg^2+^ ions that depends upon their interactions and affinities, cause metal ions to be absorbed from the environment (Mathivanan et al., 2021). The water-soluble glycopolymers give EPS special rheological and physicochemical characteristics that enable biosorption of metals. Moreover, compared to homopolysaccharides, heteropolysaccharides are more effective because they include unique functional groups (Jindal et al., 2022). They provide the EPS with its polyanionic nature, which facilitates the removal of the positively charged metal ions found in waste products.

Microbial EPS remove pollutants through multiple mechanisms, including ion exchange, complexation, microprecipitation, electrostatic attraction, and hydrophobic interaction. Functional groups such as carboxyl, hydroxyl, amino, phosphate, and sulfate groups present in EPS serve as active binding sites for heavy metals and organic contaminants (Flemming and Wingender, 2010; Gupta and Diwan, 2017). FTIR studies have demonstrated shifts in characteristic peaks corresponding to these functional groups after pollutant adsorption, while XPS analysis has confirmed metal complexation and surface adsorption process (Wang et al., 2023). Despite these advances, mechanistic understanding remains limited for mixed-pollutant systems, long-term stability of EPS-pollutant complexes, and field-scale applications, highlighting the need for further spectroscopic and pilot-scale investigations.

### 4.1 EPS-based wastewater treatment

EPS has shown great potential for removing organic and inorganic waste from water bodies. Their ability to absorb and adsorb materials, combined with their flocculation properties, makes EPS highly effective in water treatment processes. The key mechanisms behind biosorption include adsorption, absorption, intracellular and extracellular accumulation, redox reactions, precipitation, ion exchange, and surface complexation. Some recent studies have focused on the application of microbial EPS from particular species, such as *Pseudomonas pseudoalcaligenes, Bacillus mucilaginosus, Klebsiella terrigena, Exiguobacterium acetylium*, and *Staphylococcus aureus* to treat contaminated water (Siddharth et al., 2021). For example, researchers (Ferasat et al., 2020) demonstrated that EPS from *Bacillus* species were more effective than traditional coagulants for removing the kaolin turbidity from raw water. While aluminum sulfate and ferric sulfate achieved removal rates of 95 and 96%, respectively, the EPS removed 86% of the turbidity without leaving harmful aluminum or ferric ion residues. In the study by Siddharth et al. (2021), EPS from *Bacillus licheniformis*, when combined with calcium chloride, attained a 95.6% reduction in turbidity and a 61.2% reduction in chemical oxygen demand of the wastewater. Similarly, *Exiguobacterium acetylium*, derived from EPS, reduced the turbidity by 95.6% in the river water at a dosage of just 10 mg/L. Hence, it is suggested that EPS from various bacterial species could be nontoxic and thus serve as a sustainable alternative to conventional coagulants, such as alum, for water purification (Akins et al., 2020). Furthermore, some bacterial strains have been reported to effectively remove organic pollutants from river water, including *Pseudomonas putida* and *Pseudomonas plecoglossicida*, making them key players in cleaning polluted aquatic environments (Rasheed et al., 2020). Also, the non-toxic and biodegradable nature of microbial EPS further makes them far more approachable to use as a natural water treatment solution. The EPS from *Bacillus mucilaginosus* has been successfully used as a bioflocculant in the treatment of starch and swine wastewater, achieving removal rates of 85 and 91%, respectively. This bioflocculant has also proven effective in treating wastewater from breweries, pharmaceutical operations, and domestic sources with removal efficiencies of 93.6, 88.4, and 93.3%, respectively (Cheng et al., 2020).

### 4.2 EPS-based terrestrial waste treatment

Some pollutants, such as pesticides, hydrocarbons, and polycyclic aromatic hydrocarbons, harm our terrestrial environment. Several methods, including activated carbon filtration, evaporation, chemical precipitation, ion exchange, and electrochemical deposition, are used to remove these contaminants and restore soil (Duan et al., 2020). During bioremediation, biofilms that are composed of microbial populations encapsulated in an EPS matrix are essential for immobilizing pollutants. The success of *in situ* bioremediation relies on the use of mixed biofilm cultures, their pollutant-degradation abilities, and the optimization of environmental conditions to enhance microbial movement (Sivaperumal et al., 2020). Bacterial polysaccharides in biofilms have been found to bind cations and promote ion uptake, making them useful for bioremediation. The effectiveness of biofilms is attributed to their large microbial biomass, their ability to immobilize contaminants, enhanced rate of gene transfer, and strong chemotactic responses. For example, EPS from *Aspergillus niger* and *Zoogloea* spp. assists in breaking down pyrene in polluted soils, whereas EPS from *Acidithiobacillus ferrooxidans* aids in extracting metals from sulfide minerals. In the stainless steel industry, *Bacillus cereus* is used for bioremediation because of its bio-corrosion properties (Siddharth et al., 2021). EPS produced by *Nitzschia curvilineata* aids in soil decontamination by binding pollutants and restricting their movement, thereby accelerating soil recovery. Similarly, EPS from *Rhizobium tropici* enhances the capacity of soil to trap and immobilize contaminants, improving overall decontamination efficiency (Larson et al., 2016). Additionally, diatoms contribute to the removal of pollutants from coastal soils through EPS production, which helps immobilize harmful substances and mitigates their environmental impact (Hope et al., 2020).

## 5 Strategies for the use of EPS in bioremediation

### 5.1 EPS mediated biosorption and bioaccumulation of heavy metals

**Figure 2:**
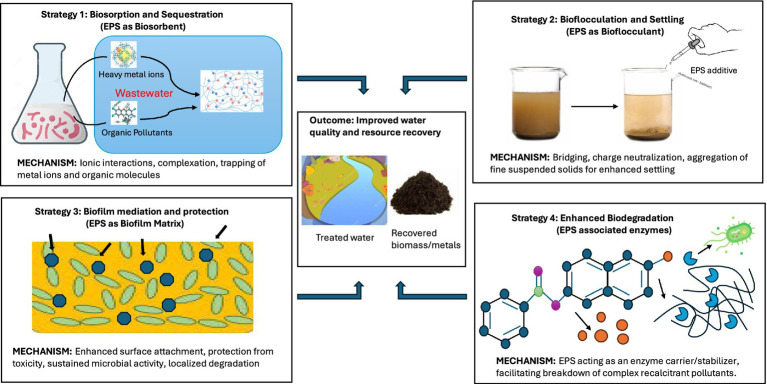
Microbial EPS contribute to wastewater treatment through biosorption, biofilm formation, biofiltration, bioflocculation, and coagulation, enabling effective removal of heavy metals, dye, hydrocarbon, and other contaminants.

The mining industry generates massive amounts of metal waste. Extracellular polysaccharides produced by bacteria have demonstrated a high level of efficacy in environmental remediation for eliminating hazardous heavy metals. This process relies on an ion-exchange mechanism or the interaction of positively charged metal ions with polyanionic bacterial extracellular polymers (Pande et al., 2022). The size, ionic characteristics, and charge activity of the metals affect how anionic bacterial EPSs interact with single or multiple metal ion solutions. Columns containing highly anionic EPS-producing halophilic bacterial strains, either immobilized or attached to suitable carriers, are used as adsorbent materials in large-scale water purification treatments (Gupta and Diwan, 2017). Strict control of pH, temperature, ionic strength, inorganic and organic ligands, and other physicochemical factors is necessary to achieve optimal outcomes (Banerjee et al., 2021). EPS-mediated biosorption also enables bacteria to utilize inorganic ions as components of their metabolism. The acetyl groups in the EPS are probably responsible for this process as they provide more electron-donating groups close to the binding sites, making it possible for bigger metal ions to attach more firmly (Li et al., 2024). EPS’s capacity to reduce the stress that heavy metals and osmotic salts like sodium cause on microorganisms also shows promise for reducing salt stress in plants that thrive in toxic or salty environments. The quality of biofertilizer can be improved, and plant resistance to toxicity and salinity can be increased by adding pure EPS or EPS-producing microorganisms (Saha et al., 2020). Neutral carbohydrates, particularly rhamnose, and possibly uronic acids, are abundant in thermophilic EPS, which makes them highly promising for use in bioleaching, metal immobilization (such as chromium and cadmium), and bio-emulsification (Banerjee et al., 2021). Initially, it was believed that heavy metals remained in their toxic state within microbial systems. However, heavy metals can be prevented from entering the biological system by converting them into less harmful or immobilized forms through biological interactions facilitated by both active processes (such as bioleaching, bioaccumulation, and toxic metal reduction) and passive processes (such as biosorption) (Zheng et al., 2024). Researchers (Kalita and Joshi, 2017) investigated that *Pseudomonas* sp. W6, isolated from a hot spring, showed high lead biosorption, removing 65% in batch and 61.2% in column studies, outperforming other strains. Its exopolysaccharide facilitated lead binding, indicating strong potential for bioremediating lead-contaminated wastewater. Ahmed et al. (2021) demonstrated that four hydrothermal vent bacterial strains (*Exiguobacterium, Mammaliicoccus, Micrococcus*, and *Jeotgalicoccus*) showed high heavy metal tolerance, EPS production, and effective biosorption, with removal efficiencies of Arsenic (83%), Cadmium (95%), Copper (94%), and Nickel (89%). Thus, for the removal of heavy metals from industrial wastewater, bacterial EPS provides a useful substitute for conventional physical and chemical approaches (Figure 2).

### 5.2 Utilization of EPS for biofilter applications

When using biological techniques to remove nutrients from wastewater treatment facilities, it is necessary to have microbial strains that can efficiently remove carbon or important elements like phosphorus and nitrogen, which are frequently immobilized in agar beads or biofilms. Airlift suspensions, trickling beds, packed beds, fluidized beds, upflow anaerobic sludge blankets, and rotating biofilms are just a few of the biofilm-based reactor designs that have been created and tested (Espinosa-Ortiz et al., 2023). The EPS material used in these systems, their filters usually contain colonies of native microbial communities, each of which contributes to the filtration process. This immobilization technique permits cell growth, retention, and ongoing activity. The characteristics and dynamics of the EPS produced in these systems are crucial for the formation of biofilms and have a direct effect on the effectiveness of biofiltration (Lago et al., 2024). By forming biofilms, microbes enclosed in the EPS layer biochemically oxidize biodegradable organic molecules, enabling in situ wastewater filtering. Additional special benefits of EPS-enclosed biofilms include enhanced gene transfer rates, microniches with gradients in oxygen and nutrient levels, and cooperative metabolism for substrate exchange. Diverse, complex microbial communities thrive in these conditions, which allow them to carry out specific metabolic or biochemical activities that improve the breakdown of organic and inorganic substances in wastewater that are difficult to break down or readily degradable (Decho and Gutierrez, 2017). Furthermore, EPS can mitigate the negative effects of toxic substances on microbial biofilters and can serve as a carbon or energy source for microbes when nutrients are lacking (Hasan et al., 2024). Mielcarek et al. (2020) found that using citric acid as a carbon source in a post-denitrification biofilm reactor produced the thinnest biofilm and lowest biomass, 53 and 61% lower than acetic acid and waste beer, respectively. The residue based biofilters removed biodegradable emerging organic contaminants (EOC) like roxithromycin (80%) and sulfamethoxazole (76%) more effectively at higher hydraulic loading rates (HLR) by enhancing biofilm activity and diversity, while lower HLRs fostered a stable microbial community with keystone species like *Bacillus*, supporting co-metabolism for removing persistent contaminants, including 17α-ethinylestradiol (48%) and sulfathiazole (37%), highlighting microbial adaptability for optimized EOC removal in water treatment (Bai et al., 2024). These promising results highlight EPS’s growing potential as a low-maintenance, cost-effective, and efficient support material for biofilm-based bioremediation.

### 5.3 Extremophilic EPS for bioremediation process

Rapid industrialization has led to a large rise in the discharge of solid waste and hazardous wastewater, two main sources of environmental contamination that have a permanent impact on the earth. Mixing, sedimentation, filtration, and chlorine disinfection are examples of traditional wastewater treatment techniques that are expensive and often produce secondary pollutants (Ahmed et al., 2021). Biological therapies usually use aerobic thermophilic bioreactors and anaerobic sludge bed or enlarged granular sludge bed reactors (Cruz-Salomón et al., 2019). These techniques are highly effective at eliminating harmful, persistent materials without creating new pollutants. While anaerobic granules have been investigated since the 1980s and aerobic granules have just recently come to light, activated sludge flocs have been the subject of intensive research and application for more than a century (Yeasmin et al., 2023). Nevertheless, there are certain drawbacks to both aerobic and biological anaerobic treatments, including reduced aggregate formation and increased sludge generation. Many bacterial EPS biopolymers are formed by extremophilic bacteria such as *Bacillus stearothermophilus* (thermophiles)*, Thiobacillus prosperus, T. acidophilus* (acidophiles)*, Listeria monocytogenes, Yersinia enterocolitica* (psychrophiles), *Gymnoascus halophilus, Aspergillus penicillioides* (halophiles), and prior research has demonstrated their promise in wastewater treatment (Haripriyaa and Suthindhiran, 2024). Most EPS, especially those from extremophiles, are polyanionic due to the presence of uronic acids or inorganic phosphate or sulfate residues, which is the main reason for their usage in bioremediation (Ibrahim et al., 2022). In environmental bioremediation, the use of EPS-enriched biofilms for (a) biofiltration (Mielcarek et al., 2020), (b) flocculation (An et al., 2023), coagulation (Mburu et al., 2025), and (c) biosorption and bioaccumulation (Zheng et al., 2024) of heavy metals in biofilms is an important method for utilizing extremophilic bacterial EPSs.

**Figure 3:**
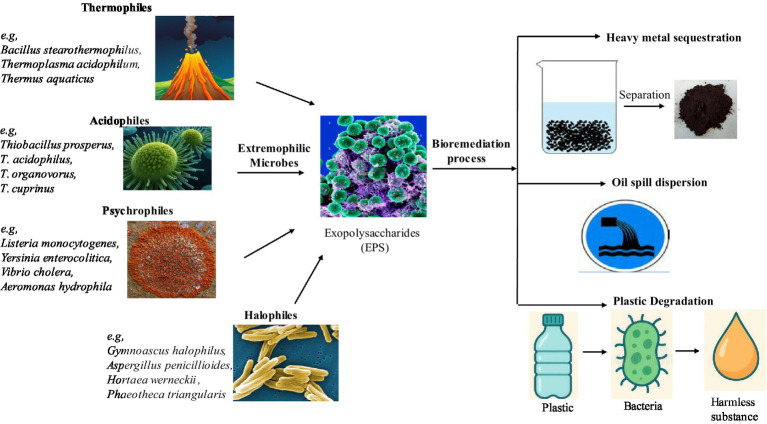
EPS produced by extremophilic microorganisms facilitate biosorption, bioaccumulation, and immobilization of heavy metals and pollutants under extreme environmental conditions, supporting efficient and sustainable bioremediation.

The microbial genes that produce EPS have already been found in a wastewater treatment plant (Wang et al., 2022). Thus, EPS-enriched extremophilic bacteria offer significant potential for bioremediation (Figure 3).

### 5.4 EPS for flocculation and coagulation

In EPS-mediated wastewater treatment, flocculation is the second-most-studied technique. It is a technique that groups tiny particles into larger clusters that settle more readily, thereby separating solids from liquids in suspensions. This technique, which depends on the formation of high molecular weight aggregates, is widely used in many different industries, such as the removal of colors from textile effluent, metal binding, paper manufacture, wastewater treatment, and algae harvesting (Ye et al., 2023). An et al. (2023) investigated the bound extracellular polymeric substances (B-EPS) isolated from *Pseudomonas* sp. XD-3 for their bioflocculation capabilities. The flocculation efficiency of B-EPS (at concentration 9–105 mg/L) ranged from 80 to 95%, under conditions with an initial pH of 4–7, kaolin concentrations of 3–7 g/L, and temperatures between 25–100 °C. Kumar et al. (2023) found that slime EPS (0.6 g/L) outperformed broth EPS in flocculation, removing 83.20% turbidity, 77.69% SS, and 76.37% COD. When combined with alum (300 mg/L) at pH 7 for 30 min, it achieved 98% removal of turbidity, 95.42% of suspended solids, and 83.08% of chemical oxygen demand (COD). Extending treatment to 4 h at pH 7 resulted in over 88% COD removal from commercial laundry wastewater. The coagulation process is crucial for lowering turbidity and eliminating dissolved organic materials. To meet stricter water quality regulations, increasing coagulant dosages is a common method to improve the removal of organic matter. This strategy enhances the effectiveness of the coagulation process in treating wastewater. Peng et al. (2024) investigated the combined use of molecular dynamics simulations, dewaterability assessment, and EPS analysis to elucidate sludge dewatering in the presence of polyaluminum chloride (PAC) and polydimethyl diallyl ammonium chloride (PDDA). PAC and PDDA promoted biopolymer aggregation via electrostatic interactions, reducing water binding. PAC outperformed PDDA in disrupting hydrogen bonds, especially in protein *β*-sheet structures, lowering EPS hydration capacity and releasing bound water, improving sludge dewaterability. Their research provides molecular-level insights into the coagulation treatment of sludge.

In another study by Mburu et al. (2025) on bacterial EPS from *Bacillus* sp., EPS treatment achieved dye (Reactive Black 5) removal of up to 85.7% and reduced COD by over 54%, with results comparable to those of chemical coagulants in textile wastewater. The EPS bioflocculants present a promising alternative to chemical coagulants, meeting environmental standards and potentially offering a safer wastewater treatment option (Figure 2; Table 2).

**Table 2:** Bioremediation mechanisms mediated by EPS.

S. no	Strategy	EPS role/Mechanism	Target pollutants	Bioremediation process	Limitations	Representative microorganism	References
1	Flocculation	Use functional groups (COOH, OH) to bridge particles, acting as natural bioflocculants	Organic matter, Heavy metals	Aggregation and sedimentation	EPS generation depends on microbial growth conditions	*Bacillus* spp.	Manognadevi et al. (2026)
2	Coagulation	Use the property of polyelectrolytes to neutralize charges and destabilize particles.	Metals, colloids	Formation of bigger aggregates	Less effective at low EPS concentration	*Klebsiella variicola B16, Azotobacter* spp., *Pseudomonas* spp.	Xia et al. (2018)
3	Biofilm formation	Creation of a structural matrix that traps pollutants and immobilizes cells	Nutrients, metals, Hydrocarbons,	Enhanced microbial degradation within biofilms	Overgrowth of biofilm may result in clogging	*Pseudomonas aeruginosa*, *Shewanella* spp.	Andersson et al. (2008)
4	Biosorption	The binding of pollutants with the help of a functional group and ion exchange	Heavy metals (Cd, Pb, Cr, Hg)	Passive adsorption into the EPS matrix	Desorption happens when there is a change in pH or ionic strength	*Arthrobacter* sp.*, Rhizobium* spp., cyanobacteria.	Pagnanelli et al. (2000)
5	Biofilter application	Stabilize microbial communities on filter media and enhance pollutant retention.	Ammonia, nitrates, and organic compounds	Continuous filtration and microbial deterioration	Accumulation of EPS may reduce permeability	Consortium of microbes	Guarin and Pagilla (2021)
6	Extremophilic EPS	Protection and binding of pollutants under extreme conditions	Heavy metals, hydrocarbons	Stabilization and sequestration under high salinity, temperature, or pH	Can be challenging	*Halomonas* spp., *Thermococcus* spp., and acidophilic archaea.	Kochhar et al. (2022)

## 6 Nanobiotechnology advances in EPS for bioremediation

Nano-biotechnology is making tremendous advances in disciplines including biology, chemistry, and physics because of its multidisciplinary nature. Nanobiotechnology has demonstrated remarkable promise in wastewater treatment, in addition to its well-established applications in medication delivery, biosensing, vaccine production, and genetic engineering (Feng et al., 2023). Biopolymeric metallic nanomaterials have attracted special scientific attention since biogenic nanoparticles have become promising options for bioremediation. In wastewater treatment research, nano-adsorbents have gained prominence due to their high surface-to-volume ratio, which increases their catalytic activity (Mondal et al., 2024). Prolonged reduction periods, intricate downstream processing, and the propensity to congregate are some of the difficulties biogenic metal nanoparticles must overcome. By acting as capping and reducing agents during the creation of metal nanoparticles, EPS biopolymers provide an answer to these problems (Parandhaman et al., 2019). By effectively interacting with metal ions, EPS provide a wastewater treatment option that targets contaminants such as pharmaceutical residues (Prasertkulsak et al., 2019), environmental metal wastes (Kalita and Joshi, 2017), textile industrial effluents (Maulana et al., 2022), and bactericidal agents (Abdalla et al., 2021). EPS biopolymers include charged functional groups and reducing groups that help them attach to other charged substances, such metal ions, they are very good at cleaning up metal pollutants in wastewater. Bacterial EPS is therefore often employed in the production of nanoparticles to help reduce the levels of hazardous heavy metals in trash that has been polluted by metals (Mahto et al., 2022). For example, mining, irrigation, and the natural weathering of rocks rich in selenium release selenium into the environment, including the very hazardous form known as selenite (SeO_3_^2−^). Selenite can be reduced and detoxified into elemental selenium nanoparticles thanks to the reducing groups in bacterial EPS (Zhang et al., 2020). Pharmaceutical waste is one of the most alarming organic pollutants in the environment due to the presence of active compounds, such as medicines and antibiotics, which cause acute water toxicity (Bilal et al., 2019). To treat organic pollutants, present in pharmaceutical wastewater, active groups of polysaccharides in microbial cell walls react with divalent metal ions in the wastewater to form metallic nanoparticles by a cation-exchange mechanism. EPS and metal ions have complementary charge interactions, which gives EPS-based biopolymeric metal nanoparticles a longer shelf life than nanoparticles made using other techniques (Ghosh et al., 2024). This makes them ideal for industrial applications. As was already indicated, EPS is used as a template for the green, eco-friendly synthesis of metallic nanoparticles. The mechanism of creating metallic nanoparticles is suggested to be two-step: first, metal ions are adsorbed onto the bacterial cell wall or a single EPS molecule, and then these ions are reduced to create metallic nanoparticles (Priyadarshini et al., 2021). Furthermore, EPS can serve as a capping agent, improving the stability of nanoparticles by inhibiting aggregation. EPS has a variety of functional groups that can act as redox pairs, including cationic (like -NH_2_, -NHCOCH_3_), anionic (like -COOH, -SO_3_, -PO_3_), and neutral (like -OH, -CH_2_, -CH_3_, -CHO) (Duan et al., 2023). According to Geng et al. (2022), the pH of the medium affects the forces that attract metal ions to these functional groups, including ionic and dipolar interactions. This results in a reduction in metal ions after the formation of an EPS-metal complex. Different functional groups in the surrounding EPS serve as anchors and stabilizers for the resultant metal nanoparticles. The basic structure of the nanoparticles is protected in the solid state, and their physical stability in solution is improved by the repulsion induced by their charged outer layer (Piacenza et al., 2018). In a brief reaction time, biogenic metal nanoparticles demonstrated strong catalytic activity to convert complex organic and aromatic contaminants or metallic wastes into non-toxic compounds.

## 7 Factor involved in biosynthesis and biodegradation of EPS

Microbial EPS production is generally influenced by chemical composition of growth medium, growth conditions, bacterial growth phase and environmental factors (Pal and Paul, 2013). EPS is generally synthesized when bacteria have excess of carbon source and limited by other important nutrients like nitrogen and oxygen (Schmid et al., 2015). A stressed environment was found to enhance EPS production in the family Enterobacteriaceae. Temperature, pH, oxygen concentration, and agitation were reported to influence EPS production in *Lactobacillus delbrueckii* subsp. *Bulgaricus* (Petry et al., 2000). In *Rhodopseudomonas acidophila*, EPS production was reported to be influenced by C:N ratio and the nature of carbon sources (Sheng et al., 2006). However, terminal electron acceptors have been shown to govern the quality and composition of EPS in *Shewanella* spp. (Pal and Paul, 2013). Interestingly, EPS production was negatively affected by nickel in the growth medium (Pal and Paul, 2013). Therefore, these factors must be taken into care when designing EPS-based bioremediation strategies.

The composition and amount of the EPS produced by microorganisms are mainly determined genetically. In several bacteria, EPS production is controlled by two-component signal transduction pathways, quorum sensing, and cyclic di-GMP (Schmid et al., 2015). Nitrogen starvation has been reported to induce EPS biosynthesis by using different components of nitrogen signaling cascade (Ruffing and Chen, 2012) and in some microorganism overexpression of genes involved in the EPS assembly (GTs, Wzx, Wzy) resulted in increased EPS yields (Schmid et al., 2015). Single-gene knockouts have also been reported to enhance the yield and chemical composition of the EPS (Núñez et al., 2009). Several c-di-GMP synthesizing diguanylate cyclase proteins having GGDEF motif as catalytic active side play crucial role in regulation of EPS-biosynthesis (Liang, 2015). Gene knockout of c-di-GMP synthases under nitrogen-limited conditions showed 57% reduction in EPS production (Ruffing and Chen, 2012).

Moreover, EPS itself is susceptible to degradation in the presence of hydrolyzing agents. Under nutrient starvation, EPS is biodegraded by its own producers and by other microorganisms. Therefore, nutrient starvation is the critical factor triggering EPS biodegradation (Zhang and Bishop, 2003). However, the chemical composition of EPS also influences its biodegradability. In microbial EPS biodegradation, the carbohydrate fraction of EPS has been reported to degrade more rapidly than the protein fraction (Zhang and Bishop, 2003). Bacterial community composition also plays a critical role in mixed culture-based biodegradation of EPS.

## 8 Application of EPS-based bioremediation and biotransformation

The application of microbial EPS for the bioremediation of industrial pollutants and heavy metals is a cost-effective strategy for waste removal. Microbial EPS have excellent metal-ion binding ability from solutions, thus making it an excellent choice for heavy metals bioremediation (Gupta and Diwan, 2017). Negatively charged moieties in EPS contribute to their anionic properties, which effectively sequester positively charged heavy metals (Muthu et al., 2017). Microbial EPS has been widely used in different forms for metal bioremediation. Major EPS-based heavy metal bioremediation strategies include homogenous consortial EPS, heterogeneous consortial EPS, immobilized EPS, dead biomass EPS, and chemically modified EPS (Gupta and Diwan, 2017). Bacterial species involved in each strategy are shown in Table 1. Microbial EPS-mediated bioremediation has been used previously to remove heavy metals from contaminated soil and water ecosystems (Kranthi Raj et al., 2018).

Marine oil spills are another serious threat to marine ecosystems. EPS-producing bacteria have previously been found to be associated with improved oil degradation capacity (Bacosa et al., 2018). Oil-degrading bacteria were potential EPS producers that, in turn, play critical roles in the emulsification and dispersion of oil droplets (Bacosa et al., 2018). Bacterial community analysis of an oil aggregate revealed an EPS-producing and oil-degrading bacterial assemblage from Gammaproteobacteria, Alphaproteobacteria, and Planktomycetes (Arnosti et al., 2016). EPS assisted oil degradation was reported in *Alteromonas* sp. strain TK-46(2) and *Halomonas* in the northern Gulf of Mexico (Gutierrez et al., 2013). Amphiphilic EPS of *Halomonas* strain effectively solubilize aromatic hydrocarbons, making them available for biodegradation by the indigenous microbial community (Gutierrez et al., 2013). Protein-rich EPS of *Alteromonas* and *Thalassospira* have also been reported to play a role in hydrocarbon degradation in oil (Bacosa et al., 2018). Therefore, these EPS producing bacteria or their purified EPS have potential for accelerating the biodegradation of aromatic hydrocarbons present in oil spillage.

Despite the enormous potential of EPS in pollutant bioremediation, EPS-based pollutants sequestration and removal still remain challenging. Enhanced volumetric production of EPS in a cost-effective manner is a major issue for downstream application of EPS in bioremediation (Nouha et al., 2018). In this regard, superior engineering strategies are continually sought to address EPS overproduction. An Increase in sugar nucleotides (EPS precursors) leading to a shift in carbon flux toward the final EPS polymer has been reported previously (Vorhölter et al., 2008). Promoter insertion in the upstream of gumC in the gum-protein operon (gumBCDEFGHIJKLM genes) of *Xanthanmonas campestris* increased xanthan biosynthesis from 66 mg cell mass to 119 mg/g cell mass (Vojnov et al., 1998). In another study, overexpression of gumD in high copy number plasmid resulted in elevated EPS production in *Xanthanmonas campestris* (Janczarek et al., 2009).

On the other hand, the engineering of functional groups present in EPS significantly alters its binding ability toward heavy metals, thereby increasing heavy-metal sequestration efficiency and rheological properties of EPS (Donati and Paoletti, 2009). Phosphorylation of cellulose obtained from *Acetobacter* has been shown to have better heavy metal sequestration (Oshima et al., 2008). However, very few studies have examined such modifications of functional groups to enhance metal ion sequestration. Another challenge in EPS-mediated biodegradation is EPS hydrophobicity, especially when EPS is used to remove organic pollutants (Nouha et al., 2018). Higher protein content or a higher protein-to-carbohydrate (P/C) ratio was reported to control EPS hydrophobicity, which in turn affects aggregate formation (Geyik et al., 2016).

Exopolysaccharides produced by various rhizobia species serve as bioemulsifiers with potential applications in hydrocarbon degradation. *Rhizobium* species also produce EPS with properties such as flocculation and metal sorption. Furthermore, EPS produced by *Klebsiella* sp. PB12 has demonstrated notable emulsifying activity with substances like toluene (66.6%), n-hexadecane (65%), olive oil (63.3%), and kerosene (50%) (Kumawat et al., 2021). Additionally, EPS produced by *Aspergillus niger* and *Zoogloea* sp. can degrade more than 80% of pyrene, a polycyclic aromatic hydrocarbon (PAH), in polluted soils. The exopolysaccharide-stabilized silver nanoparticles (AgNPs), produced by *Leucono stoclactis* KC117496, are effective in degrading the azo dyes like Methyl Orange and Congo Red (Saravanan et al., 2017). These EPS-AgNPs are synthesized by reducing Ag^+^ from AgNO₃, offering an affordable and environmentally safe approach for degrading toxic dyes. Additionally, EPS produced by bacteria such as *Bacillus firmus*, *Pseudomonas pachastrellae*, and *Bacillus cereus* has been shown to adsorb heavy metals (Li et al., 2020). Similarly, EPS produced by *Mezorhizobium loti*on glycerol-based media has been used as an emulsifying agent, with its production associated with biofilm formation, a process crucial for biosorption and biomineralization of metal ions (de Oliveira et al., 2018). Furthermore, in extreme environments, such as geothermal springs, saline lakes, and deep-sea hydrothermal vents, EPS often forms a protective layer around microbial cells and supports their survival under the harsh conditions. EPSs can be modified chemically through processes such as acetylation, methylation, carboxymethylation, and sulfonation, thereby enhancing their natural properties, broadening their biotechnological applications, and removing contaminants (Table 2).

## 9 Challenge of EPS-based bioremediation and biotransformation

EPS are essential for the bioremediation and biotransformation, as they bind with heavy metals and industrial pollutants. Thus, stabilizing them and reducing their harmful effects. However, several challenges arise in applying EPS producing microorganisms in large-scale remediation progression (Banerjee et al., 2021). One of the major challenges is reducing the bioavailability of pollutants, such as pesticides and polycyclic aromatic hydrocarbons that are strongly adsorbed onto soil particles, limiting microbial access and making degradation more challenging, especially as these contaminants age. Environmental changes, such as pH shifts during organic matter decomposition, can further destabilize pollutants and disrupt EPS-based treatments (Nkosi, 2020), potentially releasing pollutants back into the environment. Another challenge is that the EPS-producing bacteria rely on the favorable conditions to form biofilms and immobilize pollutants, but when introduced into contaminated sites, they may struggle to survive due to nutrient limitations, pH imbalances, or competition from native microbial communities. Additionally, the metabolic pathways involved in degrading pollutants are highly complex, making it difficult to optimize the performance of these microorganisms for efficient biotransformation (Zhao et al., 2024). Enzymatic interactions within the EPS matrix also need further investigation, as environmental conditions can influence enzyme activity and inhibition, affecting pollutant degradation. In environments contaminated by multiple pollutants, interactions among contaminants may enhance or hinder the bioremediation, and the precise dynamics of these interactions within EPS matrices are not yet fully understood. Water bioremediation adds further complexity, as geological and hydrological conditions significantly affect the success of EPS-based strategies in subsurface environments (Lawrence et al., 2015). Lastly, EPS stability can be compromised by environmental stress, reducing its ability to protect microbes and immobilize pollutants. This underscores the need for a deeper understanding of EPS functionality and the factors influencing its stability to achieve effective and sustainable bioremediation and biotransformation outcomes.

Despite their promising bioremediation potential, large-scale implementation of microbial EPS remains constrained by high production and downstream processing costs, variability in EPS yield and composition, and challenges associated with industrial-scale fermentation. Economic feasibility can be improved through the utilization of low-cost substrates and process optimisation strategies (More et al., 2014; Kumar et al., 2023). Regulatory approval requires evaluation of biosafety and environmental impacts. Additionally, EPS-bound pollutants may undergo desorption under changing environmental conditions, posing a risk of secondary contamination. Therefore, appropriate regeneration, stabilization, and sludge management strategies are essential for the safe and sustainable development of EPS-based bioremediation technologies (Gupta and Diwan, 2017).

## 10 Conclusion

Microbial EPS have emerged as promising eco-friendly biomaterials for remediating diverse environmental pollutants, including heavy metals, dyes, hydrocarbons, pesticides, and radionuclides. Their effectiveness is largely attributed to functional groups, such as carboxyl, hydroxyl, phosphate, sulfate, and amino groups, which facilitate adsorption, complexation, and immobilization of contaminants. Recent studies have demonstrated the significant potential of EPS-based systems for sustainable wastewater treatment and environmental restoration. However, several challenges remain, including variability in EPS composition among microbial species, limited understanding of long-term environmental behavior, difficulties in large-scale production, and insufficient field-scale validation. Future research should focus on the standardization of EPS extraction and characterization methods, pilot-scale implementation, techno-economic and life-cycle assessment, development of EPS-based composite materials, and the application of synthetic biology and metabolic engineering to enhance EPS production and functionality. Addressing these challenges will facilitate the transition of EPS-based technologies from laboratory research to practical environmental application.
